# Supplementary material for: Asymptotic expansions as control variates for deep solvers to fully-coupled forward-backward stochastic differential equations
Source: PLoS One. 2025 May 28;20(5):e0321778. doi: 10.1371/journal.pone.0321778 (PMC12118902; doi:10.1371/journal.pone.0321778)
Supplement: Attachment — Submitted filename: report.pdf [file pone.0321778.s001.pdf]

December 23, 2024

Report on
Asymptotic expansions as control variate for deep solvers to fully-coupled forward-backward stochastic differential equations

PLOS ONE

In this paper, the authors improve the numerical methods for three FBSDEs proposed by Ji et al. (2020) by incorporating the asymptotic expansion (AE) method proposed by Fujii and Takahashi (2015) as a control variate in deep learning (DL). The authors demonstrate through three numerical examples with analytical solutions that this method allows for more efficient learning compared to the original method by Ji et al. (2020), and also increases stability in machine learning predictions.

Remarks

Overall, I think this is an interesting study and it is well-organized. The proposed approximation formula provides an accurate and efficient simulation method for some FBSDEs and has the potential to be applied in various situations. However, a few areas can be improved. I offer some suggestions and questions for improvement. If these suggestions are addressed and the questions are resolved, I am confident that this study will be worthy of publication in the Plos One.

Specifically:

1 The axis labels and legends of the figures should be made clearer. For example, Figure 1 just said “with AE” and “original” but there are no explanations for these even in the main text. From the context and the caption of Algorithm 2 on page 5, it can be inferred that “original” refers to Algo. 2 of Ji et al. (2020) and “with AE” refers to Algo. 2 of Ji et al. (2020) with AE. However, it is difficult to read because Algorithm 2 in the main text and Algo. 2 of Ji et al. (2020) are mixed. Although, the horizontal axis is showed as “ computation time ” in Figure 1, vertical axis is missed. The author should write “ the value of loss function ” or “Loss_k” for the vertical axis.

2 The authors compare Algo. 2 of Ji et al. (2020) with their method (Algo. 2 of Ji et al. (2020) with AE), but there is no comparison with AE alone. They should compare three methods because, for instance, in the case of Example 1, the experiments they conducted are in very short maturity case $T = 0.1$ and low volatile case $\sigma \propto t < T$. In this case, the approximation accuracy of AE could be sufficiently high, making deep learning less meaningful (i.e., $\phi^1 \approx 0$ and $\phi^2 \approx 0$). To clarify the percentage contribution of AE and DL, the accuracy of AE should also be included. If possible, a comparison with higher-order approximations such as Fujii and Takahashi (2015) is desirable.

3 In (7) on page 3, the authors consider the FBSDE:

$$X_t^\epsilon = X_0 + \int_0^t b(s, X_s^\epsilon, \epsilon Y_s^\epsilon, \epsilon Z_s^\epsilon)\mathrm{d}s + \epsilon \int_0^t \sigma(s, X_s^\epsilon, \epsilon Y_s^\epsilon, \epsilon Z_s^\epsilon)\mathrm{d}w_s, \quad (0.1)$$

$$Y_t^\epsilon = g(X_T^\epsilon) + \int_0^t f(s, X_s^\epsilon, \epsilon Y_s^\epsilon, \epsilon Z_s^\epsilon)\mathrm{d}s + \int_0^t Z_s^\epsilon)\mathrm{d}w_s. \quad (0.2)$$

Intuitively, it should be:

$$X_t^\epsilon = X_0 + \int_0^t b(s, X_s^\epsilon, Y_s^\epsilon, Z_s^\epsilon)\mathrm{d}s + \epsilon \int_0^t \sigma(s, X_s^\epsilon, Y_s^\epsilon, Z_s^\epsilon)\mathrm{d}w_s, \quad (0.3)$$

$$Y_t^\epsilon = g(X_T^\epsilon) + \int_0^t f(s, X_s^\epsilon, Y_s^\epsilon, Z_s^\epsilon)\mathrm{d}s + \int_0^t Z_s^\epsilon \mathrm{d}w_s. \quad (0.4)$$

Indeed, the expansion becomes complex, but an approximate solution for some orders may still be achievable in this setting.

If one uses the first instead of the second one, the author may have to add some conditions and assumptions such as the impact of the arguments Y_s^ϵ and Z_s^ϵ becomes as small as possible compared to X_s^ϵ for better convergence. If my understanding is correct, in some cases, Example 1 on page 5 should use (0.3) because f has a linear term of Z. Providing necessary conditions and assumptions with theoretical justification or intuitive understanding of the difference between (0.1) and (0.3) would make it easier to comprehend.

4 In Figure 4, the yellow and gray lines overlap and are not visible. Please make adjustments to improve readability.

Minor comments

Here is a list of minor comments to help the authors to improve that paper:

1. Equation number (9) on page 5, line 11, is not referenced anywhere. May be it is Y_t^1 on page 4.
2. The ω representing the sample path is inconsistently applied to equation (1) and (4) but not to (5) and (7), which makes the readers confusing. Please standardize it.
3. Y_s in equation (7) should be Y_s^ϵ
4. Two equation numbers (9) on page 8, line 195, is not referenced anywhere. May be they are H and X, respectively.
5. Equation numbers (9) on page 8, line 209, is not referenced anywhere.

Bibliography

1. Fujii, M., and A. Takahashi (2012), "Analytical approximation for Non-linear FBSDEs with Perturbation Scheme." *International Journal of Theoretical and Applied Finance*, **15**(5), 12350034.
2. Ji., S., S. Peng, Y. Peng, and X. Zhang (2020), "Three algorithms for solving high-dimensional fully-coupled FBSDEs through deep learning." IEEE Intelligent Systems. **35**(3), 71–84.
