# Supplementary material for: Asymptotic expansions as control variates for deep solvers to fully-coupled forward-backward stochastic differential equations
Source: PLoS One. 2025 May 28;20(5):e0321778. doi: 10.1371/journal.pone.0321778 (PMC12118902; doi:10.1371/journal.pone.0321778)
Supplement: Attachment — Submitted filename: Response to reviwer 1.pdf [file pone.0321778.s002.pdf]

Response to Reviewer # 1's comments

February 6, 2025

We greatly appreciate the referee's valuable comments, which have significantly improved the quality of our paper. We have revised the manuscript in accordance with the referee's suggestions as follows:

1 To major comments

1. (comment:) *Convergence analysis of the proposed algorithm should be given.*

   (response:)

   We have added a new section titled "Stochastic Control with Prior Knowledge and FBSDE" as the second section on pages 3–5. This section shows the acceleration of convergence of our algorithm through establishing the relationship between the underlying FBSDE, stochastic control optimization, and neural networks, which is summarized as follows:

   First, Proposition 1 establishes that the solution (X, Y, Z) to the underlying coupled FBSDE (5)–(6) is obtained via the stochastic control problem (7)–(9) on page 3, which represents a continuous version of the proposed algorithm when the prior estimates $\hat{u}$ and $\hat{z}$ are set to be the processes obtained by the asymptotic expansion. Furthermore, the Universal Approximation Theorem for deep neural networks guarantees that this solution can be approximated arbitrarily well by networks with sufficient depth.

   We have also argued that when the estimates by the asymptotic expansion serve as prior knowledge for the neural networks, the control error is expected to be smaller than that in the original algorithm particularly during initial training phases, which accelerates the convergence of the algorithm as observed in Numerical Example Section.

2. (comment:) *The code of algorithm should be given in open source such as repository references.*

   (response:)

The Python code has now been published on GitHub [36]. We have added the following description on page 9, line 217:

The networks are implemented using Python pytorch, and the code is publicly available on Github [36].

2 To minor comments

1. (comment:) *Algorithm 1 missing !*

   (response:)

   In the previous version, we referred to our algorithm as "Algorithm 2" because it was based on Algorithm 2 from Ji et al. [1] combined with our asymptotic expansion method. In this revision, we have renamed it "Algorithm 1" to avoid confusion.

2. (comment:) *The notation of functions in Algo 2 such as g should be cleared.*

   (response:)

   We have added the following description to the "Input" row the Algorithm 1:

   the functions b, f, σ, g are given in (5)-(6);

3. (comment:) *Add the computation of gradient loss in Algo 2.*

4. (comment:) *The architect of NN model used should be given.*

5. (comment:) *Please see this reference: El Mouatasim, A., de Cursi, J.E.S. & Ellaia, R. Stochastic perturbation of subgradient algorithm for nonconvex deep neural networks. Comp. Appl. Math. 42, 167 (2023).*

   (response:)

   To address comments #3–#5, We have added the following description on page 8, lines 195–201:

   In the algorithm, $Y_t^{k,\pi,m}$ and $u_t^{k,\pi,m}$ are realization of $Y_t^{k,\pi}$ and $u_t^{k,\pi}$ on the m-th path respectively. Further, $\nabla Loss_k$ is computed by

$$\nabla Loss_k = \frac{1}{M}\sum_{m=1}^{M}\Big[(Y_T^{k,\pi,m} - g(X_T^{k,\pi,m}))(\nabla Y_T^{k,\pi,m} - g'(X_T^{k,\pi,m})\nabla X_T^{k,\pi,m}) + \frac{T}{N}\sum_{i=0}^{N-1}(Y_{t_{i+1}}^{k,\pi,m} - u_{t_{i+1}}^{k,\pi,m})(\nabla Y_{t_{i+1}}^{k,\pi,m} - \nabla u_{t_{i+1}}^{k,\pi,m}))\Big]. \quad (29)$$

   This algorithm can be combined with methods such as El Mouatasim et al. [35] for even greater efficiency.

Following the recommendation by [1], we use one n-dim input layer, two hidden $(n + 10)$-dim layers for both networks ϕ^1 and ϕ^2, and a m-dim output layer for ϕ^1 and a $(m \times d)$-dim output layer for ϕ^2.

Further, we have the information below on page 9, line 216:

Batch normalization is applied to each layer, and the Adam optimizer is employed.

6. (comment:) *Which programming language used for implementation? If Python give the information ' s such as Pytorch, cuda, ..*

   (response:)

   This information has been included in our response to Major Comment # 2.

3 Other major revisions

In response to other reviewers' comments:

1. We have added remarks on the computation of the asymptotic expansions (Remark 1 and Remark 2 on pages 6–7).
2. in Numerical Examples Section, we have included a comparison between neural network-based methods and methods using asymptotic expansions alone.

References

[1] Shaolin Ji, Shige Peng, Ying Peng, and Xichuan Zhang. Three algorighms for solving high-dimensional fully-coupled fbsdes through deep learning. *IEEE Intelligent Systems*, 35(3):71–84, 2020.

[35] Abdelkrim El Mouatasim, Eduardo Cursi, and Ellaia Rachid Stochastic perturbation of subgradient algorithm for nonconvex deep neural networks. *Computational and Applied Mathematics*, 42:167, 2023.

[36] Python codes on github: Deep slovers with asymptotic expansions as control variates for fully-coupled FBSDEs. `https://github.com/Makot0922/Python-Code`.
